# Diseases of the Lungs, Heart, and Kidneys

**Published:** 1893-09

**Authors:** 


*Diseases of the Lungs, Heart, and Kidneys.* By N. S. DAVIS, junr., M.D. Pp. vii., 359. Philadelphia: The F. A. DAVIS COMPANY. 1892.

This volume—"No. 14, in the Physicians' and Students' ready-reference series"—contains an elaboration of the author's notes of lectures given in the Chicago Medical College.

The subject of treatment receives especial attention, and hence the book is likely to be of service to any practitioner who may consult its pages. It contains far more information than appears on the surface, and is well worthy of careful study. Each of its sections might well serve as the nucleus of a more ambitious text-book on the respective subjects.

